# Panduratin A Derivative Protects against Cisplatin-Induced Apoptosis of Renal Proximal Tubular Cells and Kidney Injury in Mice

**DOI:** 10.3390/molecules26216642

**Published:** 2021-11-02

**Authors:** Penjai Thongnuanjan, Sirima Soodvilai, Somsak Fongsupa, Natechanok Thipboonchoo, Napason Chabang, Bamroong Munyoo, Patoomratana Tuchinda, Sunhapas Soodvilai

**Affiliations:** 1 Toxicology Graduate Program, Multidisciplinary Unit, Faculty of Science, Mahidol University, Rama VI Road, Ratchathewi, Bangkok 10400, Thailand; penjai.tho@gmail.com; 2 Research Center of Transport Protein for Medical Innovation, Department of Physiology, Faculty of Science, Mahidol University, Rama VI Road, Ratchathewi, Bangkok 10400, Thailand; Joy_nate@hotmail.com; 3 Department of Pharmaceutical Technology, College of Pharmacy, Rangsit University, Pathumthani 12000, Thailand; sirima.s@rsu.ac.th; 4 Department of Medical Technology, Faculty of Allied Health Science, Thammasat University, Pathumthani 12121, Thailand; fongsupa.mu@gmail.com; 5 School of Bioinnovation and Bio-Based Product Intelligence, Faculty of Science, Mahidol University, Rama VI Road, Ratchathewi, Bangkok 10400, Thailand; Napason.cha@mahidol.ac.th; 6 Excellent Center for Drug Discovery, Mahidol University, Rama VI Road, Ratchathewi, Bangkok 10400, Thailand; Br_411@hotmail.com (B.M.); scptcster@gmail.com (P.T.)

**Keywords:** apoptosis, chemotherapy, cyclohexanyl chalcone, kidney, oxidative stress

## Abstract

Background: Panduratin A is a bioactive cyclohexanyl chalcone exhibiting several pharmacological activities, such as anti-inflammatory, anti-oxidative, and anti-cancer activities. Recently, the nephroprotective effect of panduratin A in cisplatin (CDDP) treatment was revealed. The present study examined the potential of certain compounds derived from panduratin A to protect against CDDP-induced nephrotoxicity. Methods: Three derivatives of panduratin A (DD-217, DD-218, and DD-219) were semi-synthesized from panduratin A. We investigated the effects and corresponding mechanisms of the derivatives of panduratin A for preventing nephrotoxicity of CDDP in both immortalized human renal proximal tubular cells (RPTEC/TERT1 cells) and mice. Results: Treating the cell with 10 µM panduratin A significantly reduced the viability of RPTEC/TERT1 cells compared to control (panduratin A: 72% ± 4.85%). Interestingly, DD-217, DD-218, and DD-219 at the same concentration did not significantly affect cell viability (92% ± 8.44%, 90% ± 7.50%, and 87 ± 5.2%, respectively). Among those derivatives, DD-218 exhibited the most protective effect against CDDP-induced renal proximal tubular cell apoptosis (control: 57% ± 1.23%; DD-218: 19% ± 10.14%; DD-219: 33% ± 14.06%). The cytoprotective effect of DD-218 was mediated via decreases in CDDP-induced mitochondria dysfunction, intracellular reactive oxygen species (ROS) generation, activation of ERK1/2, and cleaved-caspase 3 and 7. In addition, DD-218 attenuated CDDP-induced nephrotoxicity by a decrease in renal injury and improved in renal dysfunction in C57BL/6 mice. Importantly, DD-218 did not attenuate the anti-cancer efficacy of CDDP in non-small-cell lung cancer cells or colon cancer cells. Conclusions: This finding suggests that DD-218, a derivative of panduratin A, holds promise as an adjuvant therapy in patients receiving CDDP.

## 1. Introduction

Cis-diamminedichloroplatinum (CDDP) is one of the most widely utilized chemotherapeutic agents for treatment of advanced ovarian cancer, testicular cancer, bladder cancer, head and neck cancer, small-cell lung cancer, non-small-cell lung cancer, cervical cancer, and breast cancer [1,2]. Major side effects of CDDP treatment include damage to kidneys, neurons, and hearing [3]. Renal proximal tubular cells are the primary target site of CDDP-induced nephrotoxicity [4,5]. Transport of CDDP into renal proximal tubular cells is mediated by organic cation transporter 2 (OCT2) [6] and copper transporter 1 [7]. The apical efflux of CDDP in renal proximal tubular cells is insufficient to avoid accumulation [8]. CDDP consequently induces DNA damage and oxidative stress [5]. These events have been reported to promote renal cell death via activation of the mitogen-activated protein kinases (MAPKs) pathway and apoptosis [9,10,11]. Therefore, a compound that suppresses oxidative stress, the MAPKs pathway, and apoptosis may serve to reduce CDDP nephrotoxicity. To date, however, there is no specific antidote to prevent kidney injury induced by CDDP [12].

*Boesenbergia rotunda* is a tropical plant that has been used as a spice and in traditional medicine [13,14]. *Boesenbergia rotunda* contains various bioactive compounds including pinostrobin, pinocembrin, and panduratin A [15]. Panduratin A is a bioactive cyclohexanyl with a molecular weight of 406.51 g/mole (C_26_H_30_O_4_). It has been reported to exhibit several pharmacological activities including anti-inflammatory [16], anti-oxidative [17], antibacterial [18,19], anti-cancer [20,21,22], anti-allergy [23], and anti-obesity effects [24]. Our recent study revealed that panduratin A attenuates CDDP-induced nephrotoxicity both in vitro and in vivo at doses of 5 µM and 50 mg/kg/day, respectively. The effect of panduratin A against CDDP-induced nephrotoxicity was mediated by the inhibition of ROS and apoptosis in renal proximal tubular cells [25]. Moreover, panduratin A itself is toxic to certain cancer cell lines and does not affect the anti-cancer activity of CDDP. Therefore, panduratin A holds promise as an adjuvant therapy for patients receiving CDDP. However, our unpublished data showed that a higher concentration of panduratin A (≥10 µM) seems to be toxic to RPTEC/TERT1 cells. In search of a compound with higher potency and less toxicity, we modified the structure of panduratin A and, then, determined the protective effect against CDDP-induced nephrotoxicity.

In this study, we investigated the protective effects and underlying mechanisms of semi-synthesized panduratin A derivatives in RPTEC/TERT1 cells and C57BL/6 mice. In addition, the effect of the panduratin A derivatives on the anti-cancer activity of CDDP was investigated in non-small-cell lung cancer cells and colon cancer cells.

## 2. Results

(±)-Panduratin A (>98% purity by HPLC) was isolated from the rhizomes of *Boesenbergia* *rotunda* as previously described [26]. Its structure was identified by direct comparison of its spectroscopic data with the literature [16,26]. Rhizomes of *Boesenbergia rotunda* were collected from Kanchanaburi, Thailand and identified by comparison with a deposit of voucher specimen (BKF No. 71812) of the Forest Herbarium, Royal Forest Department, Bangkok.

### 2.1. Synthesis of (±)-(1′R*,2′S*,6′R*)-(2-Hydroxy-4,6-Dimethoxyphenyl)[3′-Methyl-2′-(3″-Methylbut-2″-Enyl)-6-Phenylcyclohex-3′-Enyl]Methanone (DD-217)

Dimethyl sulphate (0.05 mL, 0.52 mmol, 2.1 equiv.) was added to an acetone solution (2 mL, Ar grade) of panduratin A (100 mg, 0.25 mmol) in the presence of K_2_CO_3_ (71.5 mg, 0.52 mmol, 2.1 equiv.). After refluxing for 24 h, acetone was removed by evaporation under reduced pressure. The reaction mixture was purified by chromatography on a silica gel column, eluting with 20% EtOAc-hexanes to afford DD-217 (62.0 mg, 59%; Rf = 0.53).

**Scheme 1:**
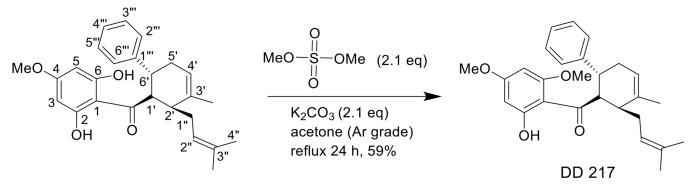
Semi-synthesis of DD-217.

**DD-217**: Pale yellow oil; UV (MeOH) λmax nm (log ε): 293 (4.32); FT-IR (ART) Vmax: 3359, 1618, 1581, 1415, 1216, 1158, 1112, 1027, 820, 757, 700 cm^−1^; ^1^H NMR (400 MHz, CDCl_3_) *δ*: 13.96 (1H, s, 2-OH), 7.20 (4H, m, H-2‴, H-3‴, H-5‴, H-6‴), 7.09 (1H, m, H-4′′′), 5.98 (1H, d, J = 2.3 Hz, H-3), 5.94 (1H, d, J = 2.3 Hz, H-5), 5.43 (1H, m, H-4′), 4.86 (1H, m, H-2″), 4.51 (1H, dd, J = 11.3, 4.6 Hz, H-1′), 3.90 (3H, s, 4-OCH_3_ or 6-OCH_3_), 3.77 (3H, s, 4-OCH_3_ or 6-OCH_3_), 3.43 (1H, m, H-6′), 2.50 (1H, m, H-2′), 2.41 (1H, m, H-5′a), 2.25 (1H, m, H-1″a), 2.06 (2H, m, H-5′b and H-1″b), 1.79 (3H, s, 3′-CH_3_), 1.50 (6H, s, 3 × H-4″ and 3″-CH_3_); ^13^C NMR (100 MHz, CDCl_3_): *δ*: 206.2 (C=O), 168.0 (C-4), 162.0 (C-2), 165.3 (C-6), 147.1 (C-1‴), 137.2 (C-3′), 131.8 (C-3”), 128.3 (C-3‴ and C-5‴), 127.0 (C-2‴ and C-6‴), 125.5 (C-4‴), 124.1 (C-2″), 120.9 (C-4′), 106.6 (C-1), 90.8 (C-5), 93.8 (C-3), 55.6 (4-OCH_3_ or 6-OCH_3_), 55.4 (4-OCH_3_ or 6-OCH_3_), 54.1 (C-1′), 42.5 (C-2′), 37.1 (C-6′), 35.8 (C-5′), 28.9 (C-1″), 25.6 (C-4″), 22.9 (3′-CH_3_), 17.9 (3″-CH_3_); ESI-MS m/z (rel. int.): 421 (37), 181 (100); ESI-MS m/z: 421.2357 [M + H]^+^ (calcd. for C_27_H_33_ O_4_ 421.2379) (Scheme 1).

### 2.2. Synthesis of (±)-(1′R*,2′S*,6′R*)-(2,6-Diacetoxy-4-Methoxyphenyl)[3′-Methyl-2′-(3″-Methylbut-2″-Enyl)-6-Phenylcyclohex-3′-Enyl]Methanone (DD-218) and (±)-(1′R*,2′S*,6′R*)-(6-Acetoxy-2-Hydroxy-4-Methoxyphenyl)[3′-Methyl-2′-(3″-Methylbut-2″-Enyl)-6-Phenylcyclohex-3′-Enyl]Methanone (DD-219)

Acetyl chloride (0.04 mL, 0.57 mmol, 2.1 equiv.) was added to a dried CH_2_Cl_2_ solution (2 mL) of panduratin A (108.8 mg, 0.27 mmol) in the presence of a catalytic amount of 4-dimethylaminopyridine (DMAP). The reaction mixture was left stirring at room temperature overnight (17 h). Water (3 mL) was gradually added, and the mixture was, then, extracted with CH_2_Cl_2_ (3 × 20 mL). Purification was performed by chromatography on a silica gel column, eluting with 30% EtOAc-hexanes to provide two separated compounds, DD-219 (83.9 mg, 70%; Rf = 0.55) and DD-218 (26.8 mg, 20%; Rf = 0.47), respectively.

**DD-218**: Pale yellow oil; UV (MeOH) λmax nm (log ε): 279 (3.99); FT-IR (ART) Vmax: 1776, 1618, 1573, 1426, 1368,1182, 1143, 1051, 882, 839, 757, 700 cm^−1^; ^1^H NMR (400 MHz, CDCl_3_) *δ*: 7.20 (4H, m, H-2‴, H-3‴, H-5‴ and H-6‴), 7.13 (1H, m, H-4‴), 6.25 (2H, s, H-3 and H-5), 5.41 (1H, m, H-4′), 5.05 (1H, m, H-2″), 3.79 (3H, s, 4-OCH_3_), 3.60 (1H, dd, J = 10.4, 3.6 Hz, H-1′), 3.40 (1H, m, H-6′), 2.46 (1H, m, H-2′), 2.29 (3H, m, H-1″a and 2 × H-5′), 2.20 (6H, s, 2-OCOCH_3_ and 6-OCOCH_3_), 2.08 (1H, m, H-1″b), 1.73 (3H, s, 3′-CH_3_), 1.65 (3H, s, 3″-CH_3_ or 3 × H-4″), 1.58 (3H, s, 3″-CH_3_ or 3 × H-4″). ^13^C NMR (100 MHz, CDCl3): *δ*:199.1 (C=O), 168.8 (2 × OCOCH_3_), 161.0 (C-4), 150.3 (C-2 and C-6), 146.0 (C-1‴), 137.8 (C-3′), 131.6 (C-3″), 128.3 (C-3‴ and C-5‴), 127.4 (C-2‴ and C-6‴), 125.9 (C-4‴), 124.0 (C-2″), 120.7 (C-4′), 119.2 (C-1), 107.4 (C-3 and C-5), 57.0 (4-OCH_3_), 55.8 (C-1′), 41.0 (C-2′), 37.6 (C-6′), 34.6 (C-5′), 29.0 (C-1″), 25.9 (C-4″), 23.3 (3′-CH_3_), 21.0 (2-OCOCH_3_ and 6-OCOCH_3_), 18.0 (3″-CH_3_). ESI-MS m/z (rel. int.): 491 (18), 449 (16), 251 (100), 209 (99), 167 (14). ESI-MS m/z: 491.2404 [M + H]^+^ (calcd. for C_30_H_35_O_6_, 491.2434).

**Scheme 2:**
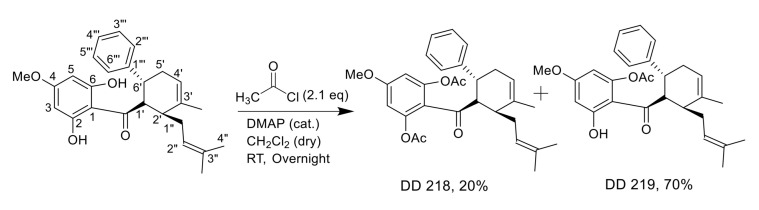
Semi-synthesis of DD-218 and DD-218.

**DD-219**: Colorless crystals; m.p. 88.2–89.6 oC (EtOH); UV (MeOH) λmax nm (log ε): 288 (4.21); FT-IR (ART) Vmax: 3358, 1776, 1622, 1423, 1372, 1184, 1065, 839, 700 cm^−1^; ^1^H NMR (400 MHz, CDCl3) *δ*: 13.35 (1H, s, 2-OH), 7.14 (2H, m, H-2‴, H-6‴), 7.04 (3H, m, H-3‴, H-4‴, H-5‴), 6.17 (1H, d, J = 2.6 Hz, H-3), 6.10 (1H, d, J = 2.6 Hz, H-5), 5.38 (1H, m, H-4′), 4.73 (1H, m, H-2″), 4.03 (1H, dd, J = 11.4, 4.5 Hz, H-1′), 3.69 (3H, s, 4-OCH_3_), 3.35 (1H, m, H-6′), 2.41 (1H, m, H-2′), 2.35 (1H, m, H-5′a), 2.28 (3H, s, 6-OCOCH_3_), 2.19 (1H, m, H-1″a), 2.01 (1H, m, H-1″b), 1.94 (1H, m, H-5′b), 1.79 (3H, s, 3′-CH_3_), 1.50 (6H, s, 3″-CH_3_ and 3 × H-4″); ^13^C NMR (100 MHz, CDCl_3_): *δ*: 204.9 (C=O), 168.4 (OCOCH_3_), 167.1 (C-4), 164.4 (C-2), 151.7 (C-6), 146.2 (C-1‴), 137.0 (C-3′), 132.4 (C-3″), 128.5 (C-3‴ and C-5‴), 126.9 (C-2‴ and C-6‴), 125.8 (C-4‴), 123.7 (C-2″), 121.4 (C-4′), 108.7 (C-1), 103.1 (C-5), 99.3 (C-3), 55.6 (4-OCH_3_), 54.3 (C-1′), 43.2 (C-2′), 37.0 (C-6′), 36.2 (C-5′), 28.8 (C-1″), 25.6 (C-4″), 22.6 (3′-CH_3_), 21.2 (6-OCOCH_3_), 18.0 (3″-CH_3_); ESI-MS m/z (rel. int.): 449 (17), 209 (100), 167 (66); ESI-MS m/z: [M + H]^+^ 449.2327 (calcd. for C_28_H_33_ O_5_, 449.2328) (Scheme 2).

**Figure 1:**
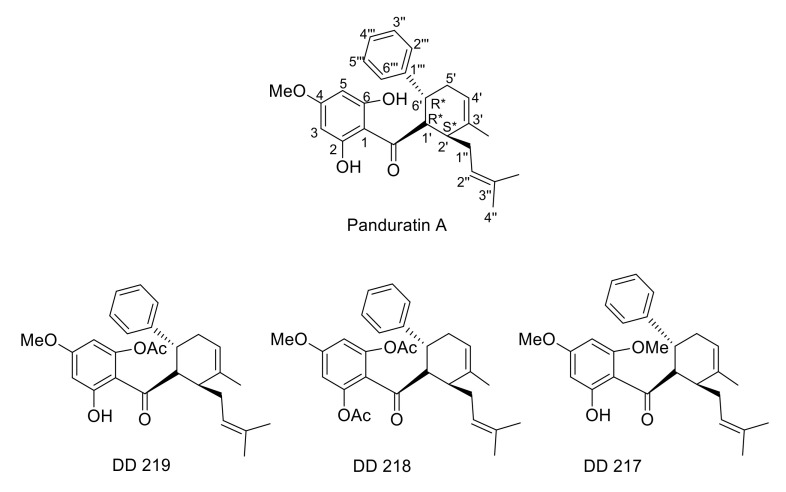
Chemical structures of panduratin A and its derivatives.

The chemical structure of panduratin A and its derivatives, DD-217, DD-218, and DD-219, are shown in Figure 1.

### 2.3. Derivatives of Panduratin A Mitigate CDDP-Induced Cytotoxicity in Human Renal Proximal Tubular Cells

**Figure 2:**
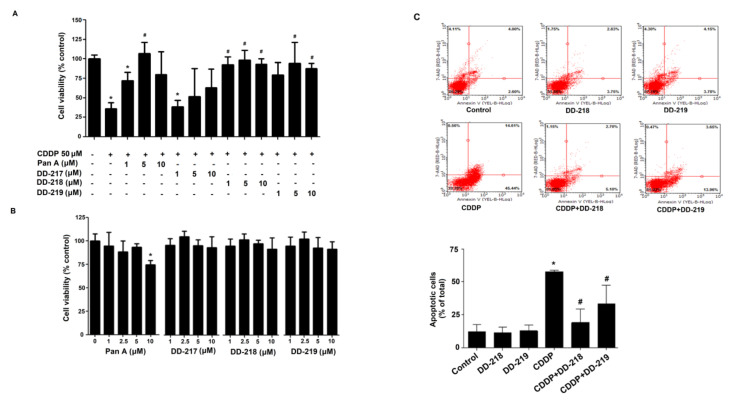
Effects of panduratin A and its derivatives on the cytotoxicity of CDDP in human renal proximal tubular cells. (**A**) RPTEC/TERT1 cell viability was measured following treatment for 72 h with: 0.1% DMSO (control): 50 µM CDDP alone; with co-incubation of panduratin A (PanA), DD-217, DD-218, DD-219 each at concentration of 1, 5, and 10 µM. (**B**) Viability of RPTEC/TERT1 cells was measured following treatment for 72 h with: 0.1% DMSO (control), panduratin A, DD-217, DD-218, DD-219 each at concentration of 1, 2.5, 5, and 10 µM. (**C**) Apoptosis of RPTEC/TERT1 cells was measured following treatment for 48 h with: 0.1% DMSO (control); 5 µM DD-218; 5 µM DD-219; 50 µM CDDP; 50 µM CDDP combined with 5 µM DD-218 (CDDP+DD-218); 50 µM CDDP combined with 5 µM DD-219 (CDDP+DD-219). Percentage of cell viability and apoptosis is shown as mean ± SD from at least 3 independent experiments: * *p* < 0.05 compared to control; ^#^ *p* < 0.05 compared to CDDP treatment.

Firstly, the protective effect of panduratin A and its derivatives on CDDP-induced toxicity was determined. RPTEC/TERT1 cells were treated with vehicle (0.1% DMSO), CDDP (50 µM) alone, or in the presence of panduratin A or its derivatives at several concentrations for 72 h. Co-treatment with panduratin A (5 µM) significantly attenuated the toxicity of CDDP. However, co-treatment with panduratin A at 10 µM showed less protective effect. Interestingly, co-treatment with DD-217 (10 µM), DD-218 (1, 5, and 10 µM), and DD-219 (5 and 10 µM) were found to protect against the cytotoxicity of CDDP (Figure 2A). We measured the effects of panduratin A and its derivatives on cell viability following 72 h incubation. Our results showed that panduratin A at 10 µM significantly reduced cell viability compared with vehicle. However, treating the cells with DD-217, DD-218, and DD-219 at 10 µM did not significantly reduce cell viability compared with vehicle-treated cells (Figure 2B). Next, we determined the effect of DD-218 and DD-219 on CDDP-induced apoptosis. As shown in Figure 2C, the treatment of cells with 50 μM CDDP for 48 h significantly increased renal apoptotic cells (57.63% ± 1.23%). The apoptotic cells induced by CDDP were significantly reduced by both DD-218 (19.06% ± 10.14%) and DD-219 (33.33% ± 14.06%). Interestingly, DD-218 showed more potent inhibition of CDDP-induced renal cell apoptosis compared to DD-219.

### 2.4. The Derivatives of Panduratin A Do Not Limit the Anti-Cancer Activity of CDDP on Cancer Cells

**Figure 3:**
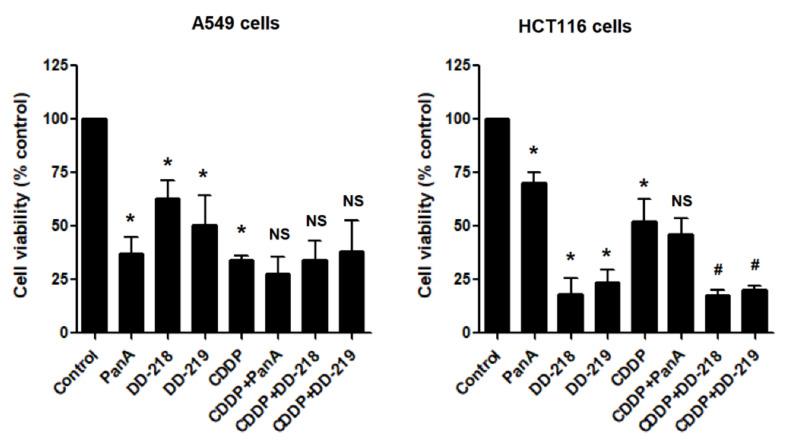
Effects of panduratin A (PanA), DD-218, and DD-219 on the cytotoxicity of CDDP on cancer cell lines. Viability of A549 cells and HCT116 cells was measured following treatment for 72 h with: 0.1% DMSO (control); 5 µM PanA; 5 µM DD-218; 5 µM DD-219; 50 µM CDDP; 50 µM CDDP in the presence of 5 µM PanA (CDDP+PanA); 5 µM DD-218 (CDDP+DD-218); 5 µM DD-219 (CDDP+DD-219). Percentage of cell viability is shown as mean + SD from at least 3 independent experiments: * *p* < 0.05 compared to control; ^#^ *p* < 0.05 compared to CDDP treatment; NS indicates non-significance compared to CDDP treatment.

The anti-cancer effectiveness of CDDP in the presence of DD-218 and DD-219 in non-small cell lung cancer (A549 cells) and colon cancer (HCT116 cells) was determined. As shown in Figure 3, the treatment of A549 cells and HCT116 cells with 50 μM CDDP for 72 h significantly reduced viability of the cells. Surprisingly, DD-218 and DD-219 by themselves significantly reduced cell viability of A549 cells and HCT116 cells. DD-218 and DD-219 did not attenuate the anti-cancer effectiveness of CDDP in the cancer cells.

### 2.5. Underlying Mechanisms of the DD-218 Derivative Reduces the Cytotoxicity of CDDP in Human Renal Proximal Tubular Cells

**Figure 4:**
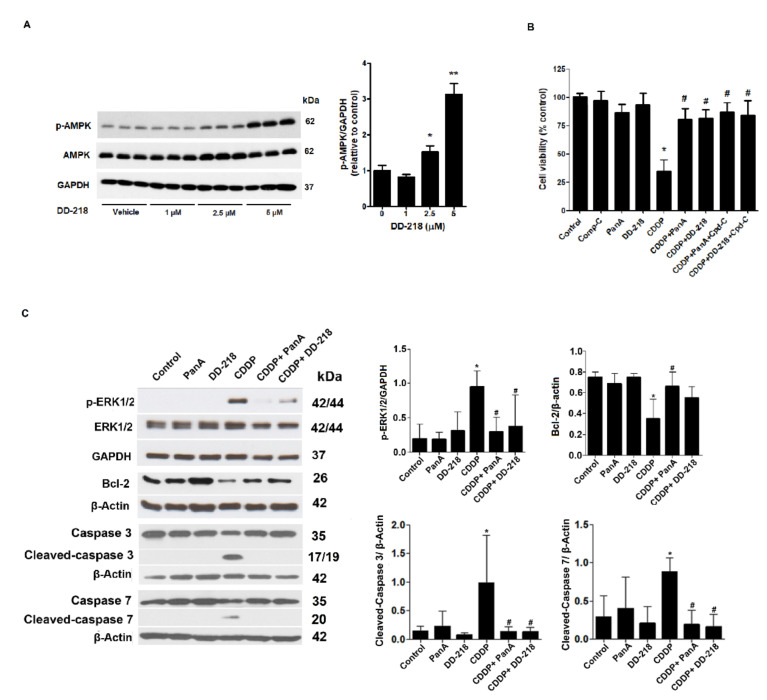
(**A**) Effect of DD-218 on AMPK expression. RPTEC/TERT1 cells were incubated for 24 h with 0.1% DMSO and DD-218 (1, 2.5, and 5 µM). (**B**) Effect of AMPK inhibitor on the protective of panduratin A (PanA) and DD-218. RPTEC/TERT1 cells were incubated with: 0.1% DMSO (control); 10 µM compound (Comp-C); 5 µM PanA; 5 µM DD-218; 50 µM CDDP; combinations as indicated. (**C**) Effects of PanA and DD-218 on expression of p-ERK1/2, Bcl-2, and cleaved-caspase 3 and cleaved-caspase 7 in renal tubular cells. RPTEC/TERT1 cells were incubated for 24 h with: 0.1% DMSO (control); 5 µM PanA; 5 µM DD-218; 50 µM CDDP in the presence of 5 µM PanA (CDDP+PanA); 50 µM CDDP in the presence of 5 µM DD-218 (CDDP+DD-218). Values are shown as mean ± SD of at least 3 independent experiments: * *p* < 0.05 compared to control, ** *p* < 0.01; ^#^ *p* < 0.05 compared to CDDP treatment.

Panduratin A has been known as a 5′ AMP-activated protein kinase (AMPK) activator. We determined whether the protective effect of DD-218 required AMPK activation. Firstly, the effect of DD-218 on AMPK activation was determined in RPTEC/TERT1 cells. The results showed that treating the cells for 24 h with DD-218 at 2.5 and 5 µM significantly increased expression of AMPK phosphorylation compared with vehicle-treated cells (Figure 4A). Next, the role of AMPK on the protective effect of DD-218 on CDDP-induced cytotoxicity was determined. Inhibition of AMPK by compound C did not attenuate the protective effect of DD-218, indicating that AMPK might not be required for the action of DD-218 (Figure 4B). The pro-apoptotic function of ERK1/2 has been implicated in CDDP-mediated apoptosis in renal cells [27]. Inhibition of ERK1/2 increases viability by inhibiting renal cell apoptosis [28]. Since DD-218 showed the most protective effect against CDDP-induced renal cell apoptosis, we selected DD-218 for subsequent experiments. First, the effect of DD-218 on CDDP-induced ERK1/2 activation was determined. RPTEC/TERT1 cells were exposed to 0.1% DMSO (control), 50 µM CDDP, 5 µM panduratin A, 5 µM DD-218 alone, or in combination with 50 µM CDDP for 24 h. As shown in Figure 4C, CDDP treatment for 24 h significantly increased the expression of phosphorylated ERK1/2. This activation was significantly reduced by panduratin A and DD-218. Next, we determined the effect of DD-218 on the expression of Bcl-2, an anti-apoptotic protein. CDDP treatment showed a significant decrease in the expression of Bcl-2. Co-treatment of the cells with CDDP and panduratin A significantly increased the expression of Bcl-2, whereas DD-218 exhibited an increasing trend in Bcl-2 expression. In addition, DD-218 inhibited the expression of cleaved-caspase 3/7 (active forms of caspase proteins) induced by CDDP.

### 2.6. DD-218 Reduces CDDP-Induced Oxidative Stress and Protects Mitochondria Function in Human Renal Proximal Tubular Cells

**Figure 5:**
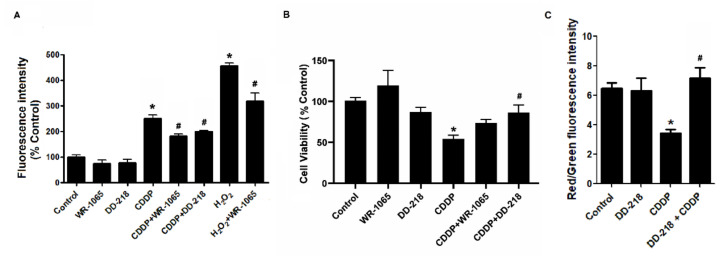
Effects of DD-218 on CDDP-induced intracellular ROS accumulation and mitochondria function in renal tubular cells. (**A**) Intracellular ROS levels of RPTEC/TERT1 cells were measured following treatment for 30 min with: 0.1% DMSO (control); 5 µM WR-1065; 5 µM DD-218; 50 µM CDDP; 50 µM CDDP combined with 5 µM WR-1065 (CDDP+WR-1065); 50 µM CDDP combined with 5 µM DD-218 (CDDP+DD-218); 1 mM H_2_O_2_; 1 mM H_2_O_2_ combined with 5 µM WR-1065. (**B**) RPTEC/TERT1 cell viability was measured following incubation for 72 h with: 0.1% DMSO (control); 5 µM WR-1065; µM DD-218; 50 µM CDDP; 50 µM CDDP combined with 5 µM WR-1065 (CDDP+WR-1065); 50 µM CDDP combined with 5 µM DD-218 (CDDP+DD-218). (**C**) Mitochondrial membrane potential was determined by measurement of red/green fluorescence ratio. Data are expressed as mean ± SD from at least 3 independent experiments: * *p* < 0.05 compared to control; ^#^ *p* < 0.05 compared to CDDP treatment.

To investigate whether DD-218 has an inhibitory effect on CDDP-induced oxidative stress in renal tubular cells, intracellular level of ROS was assessed. As shown in Figure 5A, cells exposed to 50 µM CDDP for 30 min exhibited increased intracellular ROS levels. Intracellular ROS levels were significantly reduced by co-treatment with DD-218 or WR-1065 (an active metabolite of amifostine). Next, we further compared the protective effect of WR-1065 and DD-218 on CDDP toxicity. RPTEC/TERT1 cells were incubated for 72 h with: 0.1% DMSO; 50 µM CDDP; CDDP in the presence of either 5 µM WR-1065; 5 µM DD-218. As shown in Figure 5B, CDDP treatment reduced the viability of RPTEC/TERT1 cells. Co-treatment with DD-218 significantly increased cell viability. Interestingly, co-treatment with WR-1065 showed only a slight protective effect on the cytotoxicity of CDDP. CDDP has been shown to induce mitochondria dysfunction [29]. We investigated whether DD-218 protected against mitochondria dysfunction induced by CDDP. Our results revealed that CDDP impaired mitochondrial membrane potential compared with vehicle as shown by a significant decrease in red and green fluorescence ratio of JC-1 reagent. As expected, DD-218 restored the mitochondrial membrane potential back to the basal level (Figure 5C).

### 2.7. DD-218 Does Not Inhibit Transport Function of OCT2 in Renal Proximal Tubular Cells

**Figure 6:**
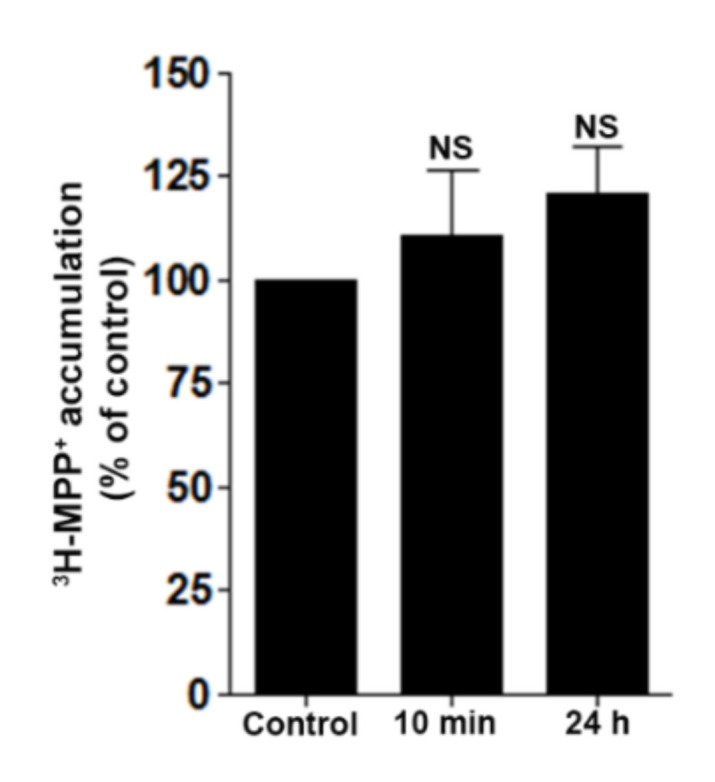
Effects of DD-218 on OCT2 function in renal tubular cells. ^3^H-MPP^+^ uptake in RPTEC/TERT1 cells was measured following incubation for 10 min and 24 h with: 0.1% DMSO (control) or 5 µM DD-218. Percentage of ^3^H-MPP^+^ uptake relative to control is shown as mean ± SD from at least 3 independent experiments: NS indicates non-significance compared to control.

OCT2 has been reported to contribute to the toxicity of CDDP in renal proximal tubular cells by increasing intracellular uptake of CDDP [6,30,31]. The protective effect of DD-218 via the inhibition of OCT2 function was determined by uptake assay of ^3^H-MPP^+^, a substrate of OCT2. OCT2-mediated ^3^H-MPP^+^ uptake in RPTEC/TERT1 cells was not significantly inhibited by incubation with 5 µM DD-218 for either 10 min or 24 h (Figure 6). Therefore, the protective effect of DD-218 might not be mediated by inhibiting OCT2-mediated intracellular uptake of CDDP.

### 2.8. DD-218 Ameliorates CDDP-Induced Nephrotoxicity in Mice

**Figure 7:**
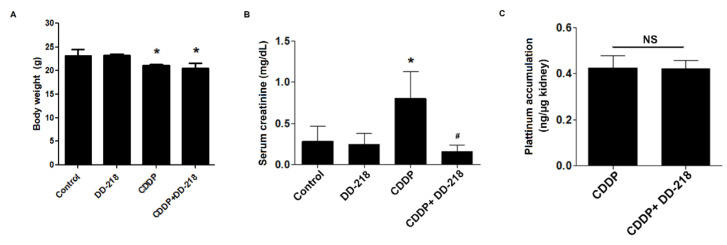
The protective effect of DD-218 on CDDP-induced nephrotoxicity. C57BL/6 mice were treated with: saline for 3 days (control) *n* = 5; 50 mg/kg/day of DD-218 for 6 days, *n* = 5; a single dose 20 mg/kg of CDDP *n* = 6; 50 mg/kg/day of DD-218 for 3 days followed by co-treatment of a single dose of 20 mg/kg of CDDP and 50 mg/kg/day of DD-218 for 3 days, *n* = 7. (**A**) Body weight of mice after treatments. (**B**) Serum creatinine. (**C**) Renal platinum accumulation. Values are expressed as means ± SD: * *p* < 0.05 compared to control; NS indicates non-significance compared to control; ^#^ *p* < 0.05 compared to CDDP treatment.

**Figure 8:**
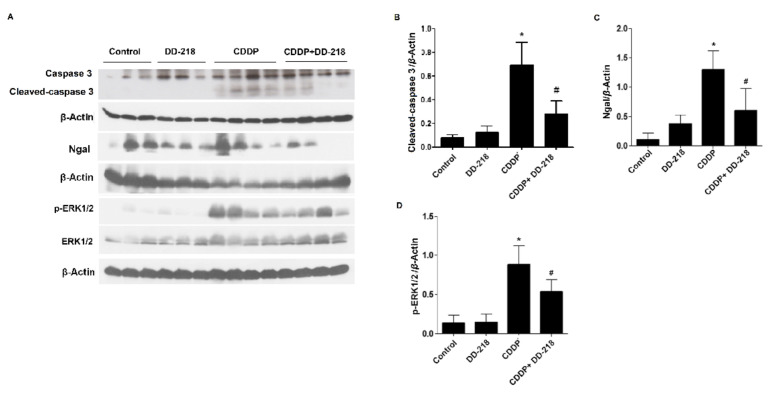
Protein expression of kidney injury markers. Representative blots (**A**) and quantitative renal expression of cleaved-caspase 3 (**B**), NGAL (**C**), and p-ERK1/2 (**D**). Mice were treated with saline for 72 h, *n* = 5; 50 mg/kg/day for 6 days of DD-218, *n* = 5; a single dose of 20 mg/kg of CDDP, *n* = 6; co-treatment with 50 mg/kg/day for 3 days of DD-218 followed by a single dose of 20 mg/kg of CDDP, *n* = 7. Values are expressed as means ± SD: * *p* < 0.05 compared to control; ^#^ *p* < 0.05 compared to CDDP treatment.

C57BL/6 mice were administered saline (via a single intraperitoneal injection), DD-218 (50 mg/kg/day orally), CDDP (20 mg/kg via a single intraperitoneal injection), or CDDP in combination with DD-218 for 72 h. Mice receiving CDDP injection had a decrease in body weight, and co-treatment with DD-218 did not restore the body weight loss induced by CDDP (Figure 7A). CDDP injection increased serum creatinine, indicating renal dysfunction. Co-administration of CDDP with DD-218 significantly improved renal function as shown by a reduction in serum creatinine (Figure 7B). To verify whether DD-218 affects CDDP accumulation in mouse kidney, platinum levels in renal tissue were determined. The results showed that DD-218 did not alter CDDP accumulation in mouse kidney (Figure 7C). CDDP-treated mice had a marked increase in NGAL expression (an early nephrotoxicity biomarker) as well as increased expressions of both active ERK1/2 and cleaved-caspase 3 in mouse kidney. DD-218 significantly decreased the expression of NGAL, p-ERK1/2, and cleaved-caspase 3 (Figure 8).

## 3. Discussion

Although CDDP is an effective chemotherapy, its effectiveness is limited due to nephrotoxicity. Development of adjuvant therapies preventing CDDP nephrotoxicity is required. The present study demonstrates the renoprotective effects of derivatives of panduratin A that do not impair the anti-cancer activity of CDDP and exhibit less cytotoxicity in normal cells than panduratin A. In this study, the RPTEC/TERT1 cells and an animal model were used to evaluate the effects and mechanisms of the derivatives on CDDP-induced nephrotoxicity.

The data revealed that DD-218, a derivative of panduratin A, showed greater attenuation of CDDP-induced toxicity than panduratin A. This notion was supported by the evidence showing 1 µM of DD-218 significantly attenuated CDDP-induced cytotoxicity, whereas panduratin A did not, as shown by 1. The data indicate that replacement the hydroxyl group in panduratin A with two groups of OAc might be responsible for the greater protective effect of DD-218. In addition, all derivatives of panduratin A tested in this study showed less cytotoxic effect than panduratin A. Replacing an OH group of panduratin A with an OAc or an OMe group reduced the toxicity. Because of the greater protective effect and lower toxicity exhibited by DD-218, we investigated the mechanisms by which DD-218 protects against CDDP-induced renal tubular cell death. Panduratin A has been reported as an activator of AMPK [24,32,33]. Here, we found that DD-218 activated AMPK in renal proximal tubular cells. However, AMPK activation did not mediate the protective effects of DD-218 and panduratin A on CDDP-induced cytotoxicity of renal proximal tubular cells. This notion is supported by data from co-treatment with a pharmacological inhibitor of AMPK, compound C. Compound C did not attenuate the protective effect of either DD-218 or panduratin A. The mechanisms responsible for CDDP–induced renal tubular cell death are complex and involve a number of interconnected factors such as ERK1/2 activation [34] and caspase activation [35]. As expected, CDDP caused renal tubular cell apoptosis via the induction of active ERK1/2, caspase 3/7 expressions. Co-treatment with DD-218 reduced the expression of both active ERK1/2 and cleaved-caspase 3/7. In cultured tubular cells, CDDP treatment has been found to decrease anti-apoptotic proteins including Bcl-2, Bcl-XL, and Mcl-1 [36,37]. Even though DD-218 exhibited the largest reduction in renal tubular cell apoptosis, the increase in anti-apoptotic protein Bcl-2 was not statistically significant in this study. A larger sample size may yield a statistically significant increase. Since panduratin A significantly increased Bcl2 expression, the data suggest that DD-218 may exert its anti-apoptotic effect via the participation of additional signaling pathways.

Oxidative stress has been reported as a significant factor that contributes to CDDP nephrotoxicity [38]. In freshly isolated renal proximal tubules, ROS formation was detectable following treatment with CDDP at a concentration of 50 µM for 30 min [29] . In this study, ROS levels were elevated after cells were treated for 30 min with 50 µM CDDP. This effect was attenuated by DD-218. The anti-oxidative effect of DD-218 was comparable to WR-1065, an active metabolite of amifostine (an anti-oxidant). Previous studies demonstrated that anti-oxidants can be an attractive option to protect against CDDP-induced nephrotoxicity [39]. Amifostine is an organic thiophosphate prodrug that serves as an antineoplastic adjuvant and cytoprotective agent in cancer chemotherapy and radiotherapy. Although amifostine was originally indicated to reduce the cumulative nephrotoxicity from CDDP in non-small-cell lung cancer, this indication was withdrawn in 2005 [40]. Amifostine is still an FDA-approved agent used for the prevention of cumulative nephrotoxicity associated with high dose CDDP in advanced ovarian cancer patients [41]. In this study, we further compared the protective effects of DD-218 and WR-1065 on CDDP toxicity in renal proximal tubular cells. We found that WR-1065 showed less cytoprotective effect than that of DD-218. These data support the contention that CDDP-induced cytotoxicity is mediated by several mechanisms, not just oxidative stress. The contention that CDDP-induced cytotoxicity is not mediated solely by oxidative stress is supported by a previous study showing that antioxidant N, N′-diphenyl-1,4-phenylenediamine (DPPD) completely inhibited CDDP-induced ROS formation but did not protect against CDDP-induce cytotoxicity in freshly isolated renal proximal tubules [29]. It has been reported that CDDP induces cytotoxicity via ROS production that influence multiple pathways. Increased ROS generation alters the mitochondrial membrane potential and triggers the apoptotic process [42]. The present study reveals that the alteration in the mitochondrial membrane potential induced by CDDP is diminished by DD-218.

Inhibition of multiple pathways might be an attractive strategy to prevent CDDP-induced nephrotoxicity. The renoprotective effects identified in this study would not be useful if the compounds impaired CDDP’s efficacy in chemotherapy. We determined that neither DD-218 nor DD-219 limited the anti-cancer effectiveness of CDDP. This finding corroborates our study reporting that panduratin A prevented CDDP-induced renal cell toxicity without altering the anti-cancer activity of CDDP [25]. Moreover, DD-218 and DD-219 are, by themselves, toxic to A549 cells and HCT116 cells. DD-218 and DD-219 exhibited a protective effect on normal cells and a toxic effect on cancer cells, suggesting the protective effect of these derivatives on renal cells is selective.

In our murine study, we revealed that co-administration of DD-218 with CDDP improved renal function and reduced renal tubule damage as shown by a decrease in both serum creatinine and the expression of NGAL. In addition, co-treatment of mice with DD-218 reduced activation of ERK1/2 induced by CDDP. This result was consistent with the data obtained from cultured human renal proximal tubular cells. Accumulation of CDDP in renal proximal tubular cells has been reported as a crucial factor determining the nephrotoxicity of CDDP [30]. However, the protective effect of DD-218 was not mediated by reducing cellular CDDP accumulation. Evidence showed that treatment of mice with DD-218 did not affect the cellular level of CDDP. DD-218 also did not inhibit the function of OCT2, a transporter responsible for CDDP uptake in renal cells [43,44]. These data support the contention that the protective effect of DD-218 is not mediated via the inhibition of CDDP transport into the renal proximal tubular cells. CDDP induces nephrotoxicity via multiple events. Thus, blocking a single injurious event may only have incomplete renoprotective effects [5]. It seems likely that DD-218 attenuates CDDP-induced nephrotoxicity via multiple events including inhibition of active ERK1/2, caspase 3, and oxidative stress.

## 4. Materials and Methods

### 4.1. Materials

Cell culture medium, fetal bovine serum (FBS), and 0.25% Trypsin EDTA solution were purchased from Gibco (Rockville, MD, USA). Penicillin and streptomycin were purchased from Invitrogen (Carlsbad, CA, USA). Transferrin, selenium, and epidermal growth factor, CDDP, 2′,7′, dichlorofluorescien diacetate (DCFH-DA), trypan blue, JC-1 dye fluorescence, and WR-1065 (active metabolite of amifostine) were purchased from Sigma (St. Louis, MO, USA). Guava Nexin Reagent (Annexin V-fluorescein isothiocyanate (FITC) and 7-Aminoactinomycin D (7-AAD) double-staining assay) were purchased from BD Biosciences (San Jose, CA, USA). Complete protease inhibitor cocktail tablet was purchased from Roche Diagnostics (North Ryde, NSW, Australia). ^3^H-1-methyl-4-phenylpyridinium (^3^H-MPP^+^; 80 µCi/mol) was purchased from Perkin Elmer (Hopkinton, MA, USA). Anti-p-ERK1/2 (Cat. No. 9102S), ERK1/2 (Cat. No. 9101S), Bcl-2 (Cat. No. 2872T), caspase 3 (Cat. No. 9662S), caspase 7 (Cat. No. 12827T), glyceraldehyde-3-phosphate dehydrogenase (GAPDH) (Cat. No. 2118S), and β-actin (Cat. No. 4970T) antibodies were obtained from Cell Signaling Technology (Danvers, MA, USA). Anti-NGAL antibody (Cat. No. STCSC-515876) was purchased from Santa Cruz Biotechnology (Santa Cruz, CA, USA). (±)Panduratin A (purity > 98%) was isolated from Boesenbergia rotunda as previously described [16,26]. All other chemicals were analytical grade and purchased from commercial sources.

### 4.2. General Experimental Procedures for Synthesis of Panduratin A Derivatives

Melting points (°C, uncorrected) were measured on a BÜCHI M-565 melting point apparatus. IR spectral data were recorded on an ART FT-IR model ALPHA spectrophotometer, while UV spectra were generated via a JASCO V-530 spectrophotometer. HR-NMR data in CDCl3 (TMS as an internal reference) were obtained with a Bruker Ascend 400 MHz spectrometer. HR-ESI-MS data were measured using a Bruker LC/MS/MS QTOF model Maxis YHR-TOF mass spectrometer. Silica gel 60 (70–230 mesh, Merck, Kenilworth, NJ, USA) was used for column chromatography. Pre-coated TLC plates 60 F254 (20 × 20 cm, Merck) were used for analytical purposes, and the bands were visualized by UV light. Solvents for extraction and chromatography were distilled at their boiling point ranges prior to use but were analytical grade for recrystallization.

### 4.3. Cell Lines and Culture

RPTEC/TERT1 cells, A549 cells, and HCT116 cells were purchased from American Type Culture Collection (Manassas, VA, USA). RPTEC/TERT1 cells were cultured in 1:1 mixture of Dulbecco’s Modified Eagle Medium and Ham’s F-12 containing 10 ng/mL recombinant human epidermal growth factor, 5 µg/mL human transferrin, 5 µg/mL insulin, 25 ng/mL hydrocortisone, 8.65 ng/mL sodium selenite, 100 U/mL penicillin, and 100 µg/mL streptomycin (pH 7.4 with HCl). A549 cells and HCT116 cells were cultured in RPMI-1640 medium and DMEM, respectively, supplemented with 10% FBS, 100 U/mL penicillin and 100 mg/mL streptomycin. All cells were incubated at 37 °C in a humidified atmosphere of 5% CO_2_ and 95% O_2_.

### 4.4. Animal

Male C57BL/6 mice (8 weeks old) were obtained from Nomura Siam International Co, Ltd. (Bangkok, Thailand). All mouse studies were conducted according to animal experiment guidelines (protocol number: MUSC61-063-464) issued by the Institutional Animal Care and Use Committee, MUSC–IACUC, Mahidol University. The mice received food and water ad libitum and were acclimatized for at least 7 days prior to the start of the experiments. Mice were divided into four groups including: (1) control; (2) CDDP; (3) DD-218; (4) CDDP+DD-218. Two groups of mice (DD-218 and CDDP+DD-218) were pre-treated by daily gavage with 50 mg/kg of DD-218 for 72 h. On day 4, administration of saline to the mice was used as the control group. A single nephrotoxic dose of CDDP (20 mg/kg/day) was administered (intraperitoneal injection) to the non-treated mice and pre-treated mice with 50 mg/kg/day of DD-218 for 72 h represented CDDP group and CDDP+DD-218 group, respectively. For the DD-218 group, the mice were continuously treated with 50 mg/kg of DD-218 for 72 h. All mice were sacrificed 72 h after CDDP administration. Blood was collected and mouse kidneys were frozen for immunoblotting and platinum analysis.

### 4.5. Cell Viability

The viability of cells treated with CDDP was assessed with trypan blue exclusion assay by exposing the treated cells to media containing 0.2% trypan blue solution. In the present protocol, a viable cell had clear cytoplasm, whereas a nonviable cell had blue cytoplasm. The cell viability was calculated as the number of viable cells divided by the total number of cells within the grids on the hemocytometer.

### 4.6. Cell Apoptosis

The apoptotic cells were evaluated by flow cytometry using Annexin V/7-AAD double-staining analysis. Cells were treated with vehicle (0.1% DMSO) or test compounds for 48 h followed by staining the cells with Annexin V-FITC and 7-AAD in the dark for 15 min. All flow cytometric measurements were performed using a BD Accuri C6 flow cytometer purchased from Millipore (Bangkok, Thailand). A minimum of 5000 events/sample were analyzed, and apoptotic cells were counted and expressed as a percentage of total cells.

### 4.7. Intracellular ROS Accumulation

The relative level of ROS was determined by DCFH-DA assay. RPTEC/TERT1 cells were seeded in 96-well tissue culture microplates containing complete medium until cells reached 100% confluence. Medium was, then, removed from all wells and discarded. After discarding the medium, 10 µM DCFH-DA was added, and the cells were incubated in humidified atmosphere of 5% CO_2_, 95% O_2_ at 37 °C for 30 min and further treated with vehicle or test compounds for 30 min. After treatment, fluorescence was measured at excitation and emission wavelengths of 485 and 530 nm, respectively. The ROS generation of treated cells is shown as a percentage of DCF fluorescent intensity relative to control.

### 4.8. Mitochondrial Membrane Potential (JC-1) Assay

Change in mitochondrial membrane potential of RPTEC/TERT1 cells was measured using the JC-1 fluorescence staining assay. After treatment, cells were incubated with Dulbecco’s phosphate-buffered saline (DPBS) containing 20 μM of JC-1 fluorescence dye for 15 min at 37 °C. Cells were, then, washed three times with DPBS before measurement of fluorescence intensity. JC-1 fluorescence excitation was measured at 488 nm. The aggregated forms of JC-1 representing normal mitochondrial membrane potential emitted red fluorescence at 595 nm, whereas monomeric forms of JC-1 denoting the decreased mitochondrial membrane potential emitted green fluorescence at 530 nm. To determine the alteration of mitochondrial membrane potential, the ratios of fluorescence intensity between red/green channels were calculated.

### 4.9. ^3^H-MMP^+^ Uptake Assay

OCT2-mediated ^3^H-MPP^+^ uptake in RPTEC/TERT1 cells was measured according to the protocol in our previous study [45]. Briefly, confluent cell monolayers on the 24-well plates were twice washed with warm transport buffer (135 mM NaCl, 5 mM KCl, 13 mM HEPES, 2.5 mM CaCl_2_·2H_2_O, 1.2 mM MgCl_2_, 0.8 mM MgSO_4_·7H_2_O, and 28 mM D-glucose) and further incubated for 30 min. The cells were incubated with buffer containing ^3^H-MPP^+^ for 10 min followed by washing with ice-cold buffer three times. The samples were collected for measurement of ^3^H-MPP^+^ accumulation using liquid scintillation beta counter purchased from Perkin Elmer (Bangkok, Thailand).

### 4.10. Platinum Accumulation in Renal Tissue

Tissue was digested with 1% nitric acid and 1% triton X-100 for at least 1 h. The sample was, then, diluted 10-fold with 1% nitric acid. A platinum standard curve was prepared using 2.5 mg/mL of CDDP diluted in 1% nitric acid using UNICAM 989 QZ AA spectrometer by Vitech International BV (Geleen, The Netherlands). The accumulated platinum in renal tissue was calculated as ng/kidney weight in µg.

### 4.11. Immunoblotting

Proteins were extracted from homogenate mouse kidneys and RPTEC/TERT1 cells after 24 h of treatment. Samples were centrifuged at 12,000 rpm at 4 °C for 20 min. Equal amounts of cells and tissue proteins were denatured and separated by 12% SDS-PAGE. Samples were transferred to nitrocellulose membranes. Non-specific protein blocking, primary antibody incubation, and protein exposure were processed according to the protocol in our previous study [25].

### 4.12. Renal Function

Serum creatinine was determined by using a Stanbio Creatinine Liquicolor test (Boerne, TX, USA). Mouse serum was collected by centrifugation of mouse blood clot at 3000 rpm for 10 min at 4 °C. Supernatant was collected and stored at −80 °C. Mouse serum was mixed with Stanbio Creatinine Liquicolor reagent (1:10). Serum creatinine was further measured by a Blood Chemistry Analyzer (Rome, Italy).

### 4.13. Data and Statistical Analysis

Data are expressed as mean and standard deviation (mean ± SD). Differences among the groups were analyzed by a one-way ANOVA combined with the Bonferroni tests (GraphPad Prism 5.0; GraphPad Software, San Diego, CA, USA). *p* values < 0.05 were considered to be statistically significant.

## 5. Conclusions

The present study revealed that DD-218 provided a marked renoprotective effect against CDDP-induced acute nephrotoxicity by attenuating oxidative stress and cell apoptosis without altering anti-cancer effectiveness of CDDP in cell lines of non-small cell lung cancer and colon carcinoma. Compared with panduratin A, DD-218 exhibited a more potent protective effect and less cytotoxic effect in human renal proximal tubular cells. Therefore, DD-218 is a potential agent for the prevention of nephrotoxicity induced by CDDP.

## 6. Patents

The data of this study have been submitted for patent (number: 2001006264).

## Sample Availability

Samples of panduratin A and its derivatives are available from the authors.
